# How Do Consumers Living in European Capital Cities Perceive Foods with Sustainability Certificates?

**DOI:** 10.3390/foods12234215

**Published:** 2023-11-22

**Authors:** Krystyna Rejman, Joanna Kaczorowska, Ewa Halicka, Aleksandra Prandota

**Affiliations:** 1 Department of Food Market and Consumer Research, Institute of Human Nutrition Sciences, Warsaw University of Life Sciences WULS-SGGW, Nowoursynowska 159 C Street, 02-776 Warsaw, Poland; krystyna_rejman@sggw.edu.pl (K.R.); joanna_kaczorowska@sggw.edu.pl (J.K.); 2 Faculty of Human Nutrition and Consumer Sciences, Warsaw University of Life Sciences WULS-SGGW, Nowoursynowska 159 C Street, 02-776 Warsaw, Poland; aprandota@gmail.com

**Keywords:** certified foods, sustainable food choices, quality labels, Poland, Belgium, cities

## Abstract

Certification aims at ensuring food quality and safety, as well as confirming other beneficial credence attributes, such as local origin and sustainability. In order to explore the visibility and credibility of such certification labels functioning in the European Union, a study was conducted among residents of two EU Member States, Poland and Belgium. Face-to-face questionnaire-based interviews and focus group interviews were conducted among 701 adults living in Warsaw and Brussels—the capital cities of these countries. Almost 44% of Belgian respondents and 33% of Polish respondents considered certified foods as being of better quality compared to unlabeled products. Focus group interviews demonstrated that Belgian consumers had more extensive knowledge and a higher level of trust in certified foods compared to Warsaw inhabitants. Our findings suggest that certificates are moderately important factors of food choice due to the wide variety of certificates, leading to consumer confusion, a lack of label uniformity, greenwashing, limited visibility and availability at points of sale, consumer price sensitivity and the prioritization of other factors. These constraints can be alleviated by introducing food labeling standards and regulations. Increasing consumer awareness and the availability and affordability of certified foods can also boost the demand for sustainable products in the region.

## 1. Introduction

Shifting towards more sustainable food systems is one of the most challenging and urgent priorities of agrifood and nutrition policy measures worldwide. In the EU region, the development of such systems is the cornerstone of the European Green Deal, introduced in December 2019 by the European Commission. The ambition of this policy is to make Europe greenhouse-gas-emissions-neutral by 2050, without giving up on prosperity [1]. In line with the Farm to Fork (F2F) strategy, which sets out strategic objectives for EU countries to make their food systems more sustainable, products offered on the market should not only meet global standards of safety, nutritional value and quality, but also comply with global standards for sustainable food. The provision of clear information that makes it easier to follow the principles of responsible consumption patterns will benefit public health and quality of life and reduce health-related costs. Although the consumer awareness of sustainable certification schemes has increased in the past two decades, it still shows significant cross-country differences [2].

To empower consumers to make informed food choices, the European Commission is examining ways to harmonize voluntary green claims and create a sustainable labeling framework that covers, in synergy with other relevant initiatives, the nutritional, climate, environmental and social aspects of food products [3]. This process constitutes a continuation of the EU food quality policy, which was developed in the 1980s under the Common Agricultural Policy. According to estimates, more than 900 certificates, marks, claims, declarations, graphics and other quality identifiers going beyond the governmental requirements operate in the EU food sector [4,5,6,7]. The number of agricultural products and foodstuffs listed in the registers of schemes that aim to protect and promote the origins, traditions and unique characteristics of EU foods (e.g., Protected Designation of Origin—PDO, Protected Geographical Indication—PGI and Traditional Speciality Guaranteed—TSG) surpassed 1650 in 2023. Additionally, almost 2000 wines and spirit drinks meet these labeling criteria [8].

Overall, the aim of food labeling schemes is to guarantee the compliance of products and production processes with the defined standards [9] and thus provide credible information about certain aspects of the food or its production method to the end-user [4,10,11]. Certification labels are intended to lend credibility to product attributes that refer to characteristics that cannot be assessed before purchase or even after purchase, especially when the label refers to upstream processes and methods. In particular, features linked to environmental, social or ethical characteristics are not physically embedded in the product; therefore, without labels, it is difficult for buyers to consider or judge the sustainability of the product that they are buying [12,13]. Accordingly, certification is a tool to reduce information asymmetry between sellers (who wish to market their products in a credible way) and buyers, who wish to satisfy a demand for high-quality sustainable goods [14,15,16]. The elimination of uncertainty about the product’s features lowers the information pre-buying costs, which results in a higher likelihood that the certificate influences consumers’ purchase decision making [17,18].

While labeling can play an important role in encouraging healthy and sustainable food choices, several barriers limit its impact on the purchasing decision. Studies show that it is not enough for consumers to be exposed to certification labels in shops, but they need to recognize them on product packaging and recognize what they mean [19]. Consumer trust is also essential for the functioning of food labeling schemes [12,15]. In general, consumers trust third-party certification more than first-party schemes, and governmental and environmental NGO-labeled products are more credible to consumers than certificates of other food chain operators [20,21,22]. The multiplicity of certification labels covering different dimensions (environment, social well-being, origin, tradition, etc.) decreases their visibility and enhances the complexity of consumer choice [15,23,24]. This leads potential buyers to feel lost and overwhelmed by the large number and variety of certified foods [7,25,26]. The high proliferation of certification labels signaling similar criteria adds to consumer confusion and label competition, where certified products compete for buyers’ attention [17,25,27].

Based on the above insights, the aim of the study was to explore and compare

- the importance of sustainable certificates on food products;
- the visibility of sustainable labels on the packaging of food products; and
- the trust in certified foods,

in consumer groups living in capital cities in two European countries: Poland and Belgium.

## 2. Materials and Methods

### 2.1. Data Collection

Two methods of primary data compilation were utilized in order to obtain quantitative and qualitative data regarding the studied issues linked to certified foods.

In the first phase of the research, data were gathered with the use of direct, face-to-face, paper and pencil interviews (PAPIs). The structured interview questionnaire was pre-tested on a group of 15 consumers and revised before data collection. The final version of the questionnaire consisted of 35 questions and was prepared in four language versions: English, French, Dutch (for respondents living in Brussels) and Polish (for those in Warsaw). The average interview time was 20–25 min. The interviews were conducted anonymously during local community events aimed at promoting sustainable (locally produced, seasonal, artisanal, organic and certified) foods. In Poland, these events included Piknik Poznaj Dobrą Żywność (Get to Know Fine Food Picnic) and Międzynarodowy Jarmark Produktów Tradycyjnych i Regionalnych (International Fair of Traditional and Regional Food Products); in Belgium, they were the Urban BBQ and piQniQ and the Gent Smaakt (Tastes of Ghent) Festival.

In order to provide a deeper understanding of the studied phenomena, focus groups (FGs) with 6–8 participants were carried out in the second phase of data collection. During FGs, participants interact and share individual experiences, opinions and attitudes, which illuminates the variety of viewpoints held in a study population [28,29]. The FG scenario used in the study was planned and pre-tested to maximize the collection of high-quality data, as well as to make sure that the interviews did not last longer than 2 h. A pilot interview was carried out in a group of 5 people and led to the introduction of minor adjustments of the final version of the interview scenario. All group interviews—5 in Warsaw and 6 in Brussels—lasted 90–120 min each and were moderated by the same person. In Poland, they were conducted in Polish; in Belgium, they were conducted in English.

### 2.2. Sample

Altogether, 701 adults participated in the study, with 359 in Poland and 308 in Belgium. In order to select the individuals, the non-probability purposive sampling method was applied. This method was chosen due to the generally low level of recognition and understanding of sustainability labels among the population [6,30,31]. With this in mind, and in order to obtain reliable research material, it was decided to conduct a survey among people who, for personal or professional reasons, were interested in high-quality foods, including those with sustainable certificates. Therefore, the research was conducted among participants at events promoting local, regional and organically certified food in the capitals of two European countries. Additionally, statistical data for Poland and Belgium confirmed that residents of large cities tend to have higher incomes and be better educated and more open to new trends, including those related to food and consumption. The food market infrastructure in big cities is also more developed than in less urbanized areas, and a larger stream of marketing activities is directed to food buyers. Therefore, inhabitants of large cities are potentially more aware of sustainability issues and more familiar with sustainable food labeling.

In total, 330 people from Poland and 329 from Belgium took part in the quantitative survey. Extra care was taken to ensure that the gender structure of the samples was similar in both countries. Respondents were asked about 6 sociodemographic variables: gender, age, level of education, the number of people living in their household, the average monthly income per person in the household and a subjective assessment of their financial situation. They also confirmed whether they were the primary food shopper in the household (Table 1).

The qualitative study involved 42 participants, with 29 in Poland and 13 in Belgium. Gender was chosen as a criterion to differentiate groups. Altogether, 6 focus groups (FGs), with 3 male and 3 female participants, were carried out. Four of them were conducted in Poland, including two FGs with women (pw1 *n* = 6, pw2 *n* = 8) and two with men (pm1 = 7, pm2 = 8). In Belgium, one FG was made up of 6 women (bw), the other FG of 7 men (bm).

The interviewees represented different professional groups and income and education levels. Five FGs were dominated by participants with higher education. Only in one group, that of the Belgian men, did participants declare a lower level of education and have blue-collar jobs.

### 2.3. Measures and Methods

The questionnaire used in the study included 7 thematic blocks on consumer purchasing behavior, their perception of high-quality food and food quality labels and their attitudes towards food products labeled with the certificate logo. For the purpose of this article, only some of the questions were analyzed (Table A4).

The perception of certified food products as better than others in the same category was measured using a single choice question with the possibility to add a comment justifying the choice. In evaluating the visibility of certified food products (i.e., point-of-sale availability), 19 different sustainable certificate logos were shown in the survey in Poland and 20 in Belgium. Among them, there were 8 international or EU certificates, while the rest were country-specific certificates. Respondents expressed their perceptions of visibility and trustworthiness using a discrete 5-point ascending scale, with the end values anchored as 1—not available/untrustworthy and 5—highly available/very trustworthy. The evaluation principles were as follows: 1–1.5—not available/untrustworthy, >1.5–2.5—hardly available/not very trustworthy, >2.5–3.5—reasonably available/moderately trustworthy, >3.5–4.5—available/trustworthy, >4.5–5.0—very available/very trustworthy.

Such a scale was also used to determine the importance of 12 factors influencing consumer food choices (1—least important and 5—most important). In order to examine the impact of the certification labels on food choices, a closed-ended question with a non-imposing nature was asked. Respondents could choose among 4 answers: “Yes”; “It depends on the products’ type”; “No”; or “I am not able to tell”.

Qualitative data were collected during the focus group interviews according to the interview scenario, which consisted of 5 thematic blocks. In order to meet the research objectives of this paper, only some of the opinions concerning consumer buying behavior and the perception of food labels were used. Participants were asked whether they knew about and bought certified foods, and whether certification labels mattered to them when shopping. During the group interviews, different types of mock-ups as discussion stimuli were used, such as

- photos of different certification labels operating on the Belgian (20 labels) and Polish (19 labels) food markets: at first, the graphic symbol itself, and then the symbol and its name next to it;
- boards presenting a set of food products from the same product category—for example, 5 types of butter with different certification labels;
- photos of selected promotional campaigns aimed at increasing the recognition of certificates among consumers and creating a demand for certified food.

### 2.4. Data Analysis

The quantitative data analysis was performed in the Statistical Package for Social Sciences (IBM SPSS Statistics, version 25, SPSS Inc., Chicago, IL, USA). Data were initially investigated through descriptive statistics (frequency, means and cross-tabulations). For the correlation analysis, the Pearson’s non-parametric chi-square test was used; for the comparative analysis, Kruskal–Wallis’ test was used. The nominal variables were compared using Pearson’s chi-square test. For orderly variables (age and number of people per household), a non-parametric Mann–Whitney’s test was applied. A level of *p* ≤ 0.05 was considered significant. The V-Cramer test was used to determine the associations between variables. The Cramer’s V coefficient ranges from 0 to 1, and the interpretation of the strength of the relationship between the variables is as follows: V values around 0.1 indicate a weak correlation (although the result is statistically significant, the items are only weakly associated), those around 0.3 indicate a moderate correlation and those around 0.5 or higher indicate a strong correlation (the fields are strongly associated).

## 3. Results

### 3.1. The Perception and Visibility of Food Certification Labels

Every third respondent surveyed in the quantitative study living in Warsaw and 44% of the respondents from Brussels considered certificates to be a sign that the certified product is better than others in the same category (Table 2).

Statistical relationships were found between the positive perception of certified products and the sociodemographic characteristics of the respondents. Among Poles, it was linked to a higher household income level (*p* = 0.0298, Cramer’s coefficient V = 0.1776) and the age of the respondent (*p* < 0.0001, V = 0.2519). In the case of Brussels residents, those from larger households had a statistically better perception of certified products (*p* = 0.0187, V = 0.2250).

**Figure A1:**
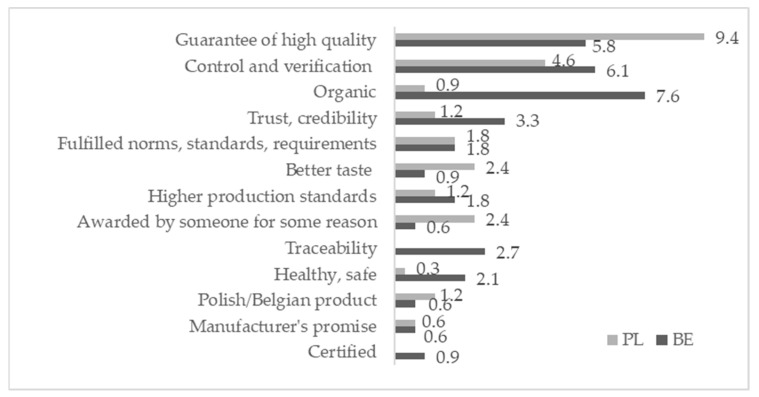
Reasons to perceive a certified product as superior (% of answers).

The comments associated with the “yes” answer gave insights into the reasons for perceiving certified products as better than others in their category (Figure A1). The advantages most frequently mentioned by Poles were a guarantee of high quality, the control and verification of production processes, a better taste and distinction for a specific reason. Belgians usually indicated “organic” production and the control and verification of production processes.

**Figure A2:**
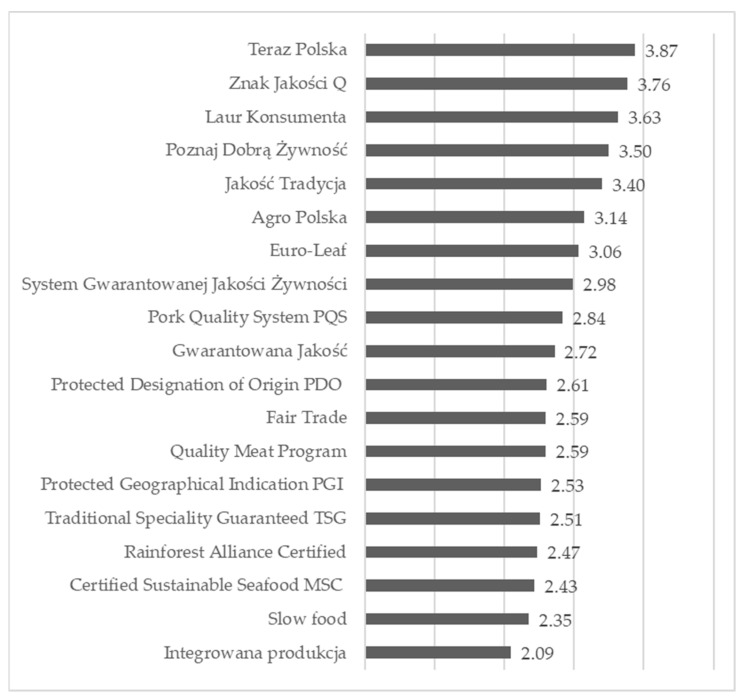
Visibility of products with a specific certification label, evaluation of Polish consumers (5-point scale, where 1—not available, 5—highly available at the points of sale). Assessment of visibility (availability at the point of sale): not available 1.0–1.5, hardly available >1.5–2.5, reasonably available >2.5–3.5, available >3.5–4.5, highly available >4.5–5.0.

**Figure A3:**
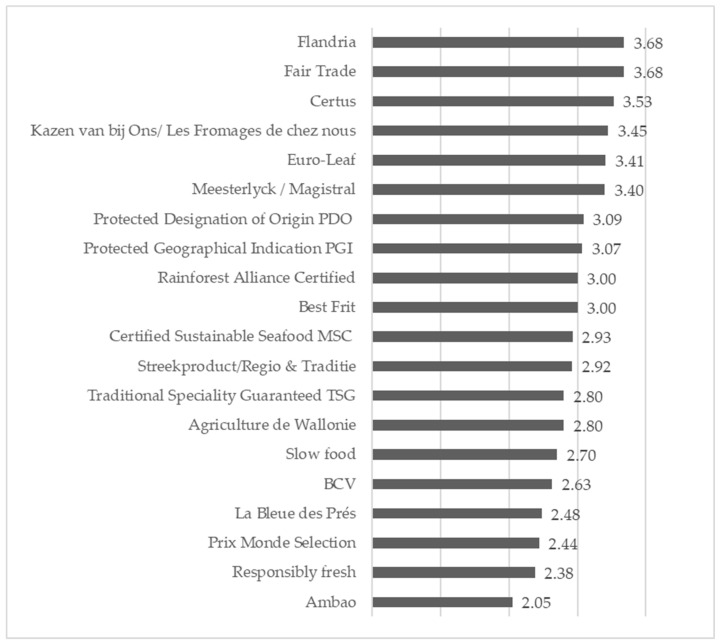
Visibility of products with a specific certification label, evaluation of Belgian consumers (5-point scale, where 1—not available, 5—highly available at the points of sale). Assessment of visibility (availability at the point of sale): not available 1.0–1.5, hardly available >1.5–2.5, reasonably available >2.5–3.5, available >3.5–4.5, highly available >4.5–5.0.

The average ratings for the visibility of 19 certificate logos among Polish respondents and 20 certificate logos among Belgium respondents were in very similar ranges in both countries. In the case of Polish respondents, the mean varied from 2.09 to 3.87; for Belgian respondents, it ranged from 2.05 to 3.68 (Figure A2 and Figure A3). In both countries, products with three certificates were assessed as available, with four as hardly available and with the rest as reasonably available (12 in Poland and 13 in Belgium), according to the adopted evaluation principles. According to Warsaw residents, the most visible products were those with three national (Polish) food certification labels: *Teraz Polska* (3.87), *Znak Jakości Q* (3.76) and Laur Konsumenta (3.63) (Figure A2). Respondents rated the visibility of these products as available. The visibility of products having international or EU certificates was lower. The highest rating was given to the visibility of products with the Euro Leaf logo among the eight considered (Table 3). The weighted average was 3.06 (reasonably available) and marked only the seventh place of these products in terms of availability at the point of sale. Moreover, the Fairtrade certificate and the three food quality certificates for regional and traditional products in the EU were assessed as reasonably available. Food products certified by the Rainforest Alliance, MSC Certified Sustainable Seafood and Slow Food were considered as hardly available.

Brussels residents indicated the highest visibility of products with national logo Flandria, international certificate Fairtrade (3.68 in both cases) and national symbol Certus (3.53) (Figure A3). It should be noted that among respondents in Brussels, the visibility of products with EU or international certificates was, in each case, higher than for respondents in Warsaw (Table 3). Significant differences between the compared groups of respondents were noticed for four logos: Euro Leaf (*p* < 0.0001), Fairtrade (*p* < 0.0001), PDO (*p* = 0.0011) and PGI (*p* = 0.0034). The greatest differences in visibility concerned the Fairtrade certificate. In Belgium, Fairtrade-certified food was rated as the second most visible (out of 20 evaluated certificates) and in Poland as the thirteenth.

The respondents’ opinions were determined by their sociodemographic characteristics. The visibility of the Fairtrade certificate in both cities depended on the level of education (Table A2). Among Warsaw residents, the perception of the availability of Euro Leaf-certified products changed with age, while, in the case of Brussels residents, a correlation was found between the visibility of the PDO and the number of people in the respondent’s household.

In the qualitative part of the research, most respondents admitted that they noticed certified food products at the points of sale on a daily basis. FG participants often associated certificates (whose symbols were shown to them) with a specific group of products—for example, *“You can usually find Rainforest Alliance Certified on coffees and teas or at the entrances of some cafes”* (Polish woman 1, pw1), *“All Parmesans are labelled with PDO sign”* (pw2), *“There are Ambao products in the store, this is my favorite mark, I love this chocolate”* (Belgian man, bm), *“Sometimes in restaurants, I choose a meal prepared only from Fairtrade products”* (Belgian woman, bw).

During the discussion, it was noted that respondents from Belgium were more aware of the existence of different national, EU and international certification schemes, while the statements of Poles mainly concerned national certifications.

### 3.2. Trust in Certified Food Products

**Figure 1:**
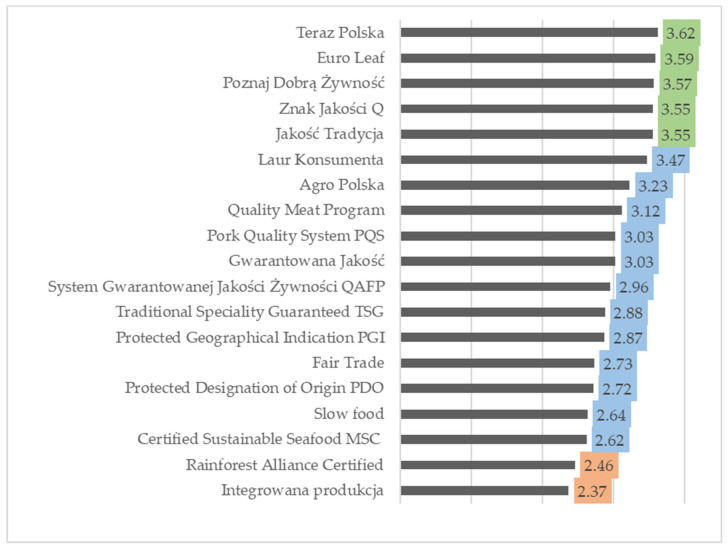
Polish respondents’ trust in food certificates (5-point scale, where 1—untrustworthy, 5—very trustworthy). Assessment of trust: untrustworthy 1.0–1.5; **not very trustworthy >1.5–2.5**; **moderately trustworthy >2.5–3.5**; **trustworthy >3.5–4.5**; very trustworthy >4.5–5.0.

**Figure 2:**
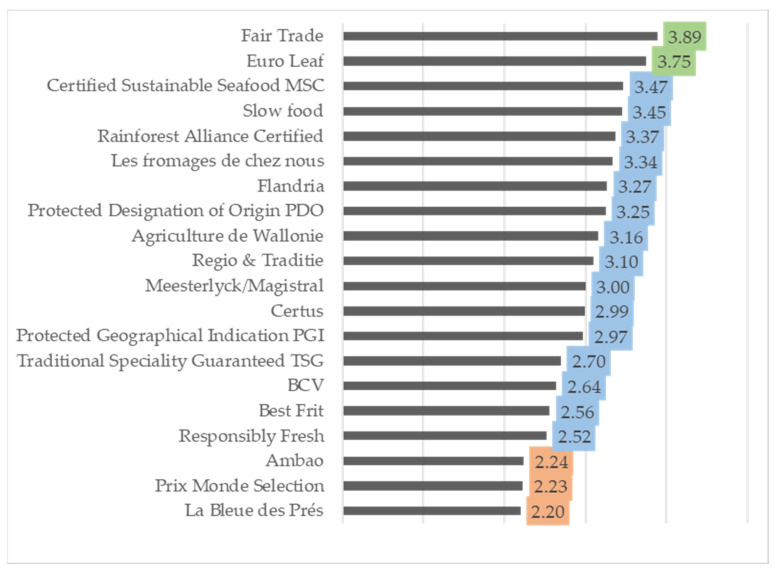
Belgian respondents’ trust in food certificates (5-point scale, where 1—untrustworthy, 5–very trustworthy). Assessment of trust: untrustworthy 1.0–1.5; **not very trustworthy >1.5–2.5**; **moderately trustworthy >2.5–3.5**; **trustworthy >3.5–4.5**; very trustworthy >4.5–5.0.

The average trust scores for the certificates presented to the study participants were in very similar ranges in both countries. The means in Polish respondents ranged from 2.37 to 3.62 (Figure 1) and they ranged from 2.20 to 3.83 in the Belgian group (Figure 2). In both countries, all certificates were classified into three out of five trust categories, i.e., trustworthy, moderately trustworthy and not very trustworthy, according to the established evaluation principles. In the Warsaw group, consumers most trusted five certificates, *Teraz Polska* (3.62), Euro Leaf (3.59), *Poznaj Dobrą Żywność* (3.57), *Znak Jakości Q* (3.55) and *Jakość Tradycja* (3.55), which they assessed as trustworthy (Figure 1). Two other labels, *Rainforest Alliance Certified* (2.46) and *Integrowana Produkcja Roślin* (2.37), were rated as not very trustworthy. This was due to the unclear communication of these certificates and their relatively low visibility at points of sale. The other labels (12) were considered moderately trustworthy. Among them, the remaining six international and EU certifications gained the lowest trust in this category.

Brussels residents declared that they trusted the Fairtrade certificate (3.89) and the Euro Leaf (3.75) the most. On the other hand, three national certificates—*La Bleue des Prés* (2.20), *Ambao* (2.24) and *Prix Monde Selection* (2.23)—were seen as the least trustworthy (mean score—not very trustworthy). The remaining 15 certificates were seen as moderately trustworthy (Figure 2).

The Belgian respondents demonstrated greater trust in international and EU food certifications than the Polish participants of our study, rating five international certificates the highest. Warsaw inhabitants recognized five certificates as trustworthy, but only one of them was international (Euro Leaf).

The respondents’ place of living had a significant influence on their level of trust in foods certified with international or EU certificates (Table 4). Among Polish respondents, trust in PGI- and PDO-certified foods depended on the level of education (Table A3). In addition, trust in PGI-certified foods depended on the age of respondents, while the household size influenced trust in Rainforest Alliance-certified products. Age also determined Belgian respondents’ trust in Slow Food certification.

The problem of limited trust in some certification schemes was also detected during the focus group interviews. Such opinions were more common among the male groups, e.g., *“I do not trust these signs because I believe that the company can place the logo like that by itself”* (pm1), *“I think the producer only has to pay for the certificate, nothing has to prove”* (bm1). According to women, international or EU certificates were more trustworthy than country-specific ones, e.g., *“If I had to choose the ones I trust from among all the signs shown, I would choose those with EU guarantees, i.e., TSG, PGI and PDO”* (pw). The main reason for respondents’ distrust of food certificates was the way in which they are awarded and the procedure that a producer has to go through to obtain the right to use them, e.g., *“There is no such label which says: we, an independent organization, which has nothing to do with the producer, certify that this product was produced in a sustainable manner”* (bm2).

Participants from both countries also noted that there were too many signs and symbols on food labels, creating confusion about their meaning, e.g., *“with a lot of time maybe you will be able to decode what all these signs mean, but another question is whether they are actually verifiable”* (pm2). Finally, it was also seen that Belgian groups attached more trust to certified foods compared to Polish respondents.

### 3.3. Influence of Certificates on Purchasing Behavior

Both Polish and Belgian respondents declared that certification logos on food products influenced their purchase decisions; however, to a large extent, this depended on the type of food (Table 5). One in five respondents declared that the certificate on the product packaging did not affect her/his purchase.

A statistical relationship between the responses given by Polish consumers and age (*p* = 0.0076, V = 0.1676), as well as the number of people in the household (*p* = 0.0172, V = 0.1851), was noted. In the Belgian group, sociodemographic characteristics did not determine the answers.

In the qualitative part of the survey, participants generally agreed that labels had a rather moderate influence on their food purchases. In the discussions with women, it was noted that even good knowledge and trust of the certificate did not always translate into their purchasing behavior. According to the male respondent groups, producers should be careful not to include an excessive number of certificates on the packaging, as this discourages the purchase of the product (Table 6).

### 3.4. Factors Influencing Consumer Food Choice

When rating the importance of 12 potential food choice factors (ascending scale from 1—least important to 5—most important), freshness emerged as the top factor for the entire sample and in both countries (Table 7).

Certificates and symbols on product packaging indicating special qualities were pointed out as important (score 4 or 5) by 36% of participants from Warsaw and 40% from Brussels. Even fewer respondents (30% and 18%, respectively) reported that they paid attention (score 4 or 5) to other symbols suggesting the uniqueness of the product.

Significant differences were found in the importance of seven factors: freshness, nutritional and caloric value, brand reputation/trust towards the producer, symbols on the packaging that indicate the distinction of a product, practical/convenient packaging, esthetical packaging and advertising. The in-depth analysis allowed us to observe a number of significant correlations in the assessment of the importance of food choice factors with the sociodemographic characteristics of the respondents (Table A1). The explanatory variables for the choice factor scores appeared to be age, net income per person, education level, subjective evaluation of material situation and the number of people in the household (Table 7).

In the qualitative part of the study, participants were asked to list the most important food attributes that they considered when choosing food (Table 8). The results showed that sensory attributes (taste, appearance, etc.), product freshness and shelf life, as well as brand reputation (or trust towards the producer), were the main factors influencing respondents’ food choices. Food product composition and nutritional value, price, packaging, country and place of origin and promotions and advertising were indicated as other important features of the food when making purchasing choices.

It was observed that FG participants did not show much interest in quality labels, symbols or certificates when selecting food. They used simplified terms for them and described certification labels as signs, marks, symbols or simply as graphics (in the quoted statements of the respondents, these terms were left unchanged). Female participants in the interviews stated that they usually did not trust certification labels when choosing food, because it is not clear what they actually certify: “*producers cleverly place on products something related to certification, distinction or quality, such as a charming red ribbon or Victory Laurel. (…) It is not clear who assessed them and what these symbols refer to*” (pw). On the other hand, male respondents noted that when choosing food, they generally did not pay attention to them due to the huge number of certification labels functioning in the food market: *“I know that there is something like that, I can see it almost on every product (…) due to the fact that there are so many of them, I stopped reacting to it”* (pm).

## 4. Discussion

Food choice is determined by a complex set of determinants that act as incentives, barriers or conditions and whose importance differs between populations. As the level of wealth increases and individual needs are more fully satisfied, the importance of price–income constraints on consumption decreases, while the number and expression of non-economic determinants grows [32]. In the context of the SDGs, factors linked to marketing and labeling are particularly relevant as the principles of a sustainable diet include choosing high-quality foods that meet credible certified standards [33,34], and considering Marine Stewardship Council (MSC), free range and Fairtrade products [35]. The rationale behind these guidelines is to increase the awareness among consumers that buying certified, high-quality foods contributes to addressing sustainable food system challenges, including climate change, food security, biodiversity, animal welfare and water scarcity. Certified food products, depending on the type of certification, fulfil the key components of sustainable food consumption linked to food and nutrient needs, food security and accessibility, well-being and health, biodiversity, the environment and climate, equity and fair trade, eco-friendliness, local and seasonal foods, cultural heritage and skills [36]. Food labeling has become part of the food system infrastructure; however, there are challenges in governing this sector [37]. Sustainable consumption is therefore a concept that goes beyond the traditional understanding of consumerism and requires responsible purchasing decisions [38]. EU inhabitants are becoming increasingly attentive to these dietary considerations, with those on higher incomes looking for food that not only meets taste expectations but is also authentic and produced in a traditional way [39].

Our study showed the moderate importance of food certification as a factor impacting consumer choice. The average rating of the influence of labels on a 5-point scale was 3.04 and 3.16 among Polish and Belgian respondents, respectively. The results of the Special Eurobarometer 473 survey involving all Member States also showed no major differences in the evaluation of the importance of a specific label ensuring quality. It was found to be a very or fairly important factor for more than 60% of respondents in all countries, with the average percentage of 75–76% in Poland and Belgium [40].

Based on both quantitative and qualitative data collected during the research, sensory attributes were the top food choice factors in the studied urban populations. According to another EU-wide study, the taste of food was the most important driver of food purchasing decisions in all Member States [41,42]. In the case of three food choice factors referring to environmental and social aspects of consumption, minimally processed food, the geographical origin of food and the personal ethics and beliefs of the consumer (in terms of religion, animal welfare, fair payment for producers), the answers of respondents from Poland and Belgium did not differ. These features were of the least importance (in that order they were indicated, in sixth, seventh and eighth place), and the percentage of declarations ranged from over 20% for geographical origin and the degree of food processing to only 12% in Poland and 10% in Belgium in the case of ethical aspects [41].

Product labeling with marks denoting some kind of award or distinction had a weaker influence on food selection decisions than certificates. This factor was rated significantly higher by Warsaw residents, probably due to the design of such marks, which are usually accompanied by a convincing inscription, e.g., Laur Konsumenta (The Consumer Laurel), and the high ethnocentrism of Poles, caused by both objective and subjective factors [43,44,45,46]. The same considerations may have been responsible for the assessment of the visibility of and trust in products certified and awarded with these symbols at points of sale. Warsaw respondents ranked six Polish certificates at the top of the visibility ranking; Brussels respondents ex aequo indicated the national Flanders certificate and the international Fairtrade certificate. In the trust ranking, Polish respondents listed the same Polish certificates highest, except for one, as Euro Leaf came in second. Belgian respondents indicated four global certificates and one EU certificate as the most trustworthy. Trust and transparency in any labeling scheme is essential for it to be meaningful and motivate change in individuals or industries [37].

The assessments of Belgian consumers were not consistent with the results of the survey among EU countries, in which national labels were much better perceived by the respondents compared to their EU counterparts [42]. In our study, both groups rated their trust in the EU organic label very highly, whereas, in the study cited above, despite being the most recognizable, it was rated worst among labels, with the level of consumer trust being quite low. Confusion has arisen over the use of the term “organic” on food products. These products can be associated with a so-called health halo. This health association with organic products is probably more related to the values attributed to organic production practices than to the food itself, as there is limited evidence to date to suggest the superior nutritional quality of organic products.

In a survey covering all EU countries [41], Belgian respondents declared significantly higher awareness of international and EU certificates compared to Polish respondents. Awareness of at least one certificate was declared by 83% of Belgians and only 44% of Poles. The awareness of Fairtrade was 68% and 9%, and that of organic farming was 39% and 29%, respectively. The results of this study support our findings as well as reflecting the effectiveness of promotional activities undertaken at a government level in both countries. In Poland, between 2013 and 2015, the Ministry of Agriculture and Rural Development implemented an intensive promotional campaign for these EU “Three Flavour Marks”, i.e., the PDO, PGI and TSG food certificates [46]. The Belgian Ministry of Foreign Affairs, Foreign Trade and Development Cooperation [47] specified targets for the demand for Fairtrade-certified products in the 2020 perspective: increased household expenditure on food with this certification; recognition of the certificate among 95% of Belgians; the offering of certified products by all major supermarket chains, etc. In this context, it is important to note opinions on the negative effects of this certification system on producers from developing countries, namely that the solutions used in the system are not compatible with free trade and free market principles [48]. Higher wages do not increase the efficiency of workers but contribute to the elimination from the market of poorer producers who are not covered by the scheme [49]. The implementation of the system has hardly any impact on farmers’ incomes and poverty reduction; a better example in this respect is the impact of Rainforest Alliance Certified [50].

Awareness, knowledge and trust are sufficient conditions for a certificate to fulfil its function as a decision aid, supporting consumers in choosing foods according to their preferences. The examples of Poland and Belgium show that effective communication campaigns can serve as a tool to raise consumer awareness and knowledge and, if other conditions are met, can boost the sales of certified sustainable products. Among additional conditions, the respondents’ financial capabilities are important. An increased demand for products that comply with the principles of sustainable consumption also requires greater environmental and consumer responsibility [51,52].

Our research identified the respondents’ limited trust in certified foods, not least because of the wide discretion in establishing labeling schemes. Indeed, different entities can certify that a product’s characteristics comply with certain criteria. In business-to-business (B2B) communication, certification is always attested by a third party, e.g., an independent certification body, a state authority, an influential industry association or a representative of a religious group [53,54]. In business-to-customer (B2C) relationship, it is also acceptable to confirm certification on the basis of a self-declaration [54,55]. Consumers are more likely to trust certification labels developed by independent organizations as well as government agencies [20,22,37]. They also often have insufficient knowledge of the principles and organizations behind certification, which can result in an effect known as “label fatigue” [56,57,58]. Since the use of self-declared sustainability claims is still loosely (or not at all) regulated in many countries, and marketers still often exhibit one or more “sins” of greenwashing, it could be useful for policymakers to provide rules/guidelines that producers should respect when stating their socio-environmental commitments, through on-pack labeling [2].

The surveyed respondents also pointed out the multitude of certifications designed to attract the buyer’s attention and induce a purchase. Some studies show that consumers can become confused and overwhelmed by the large number and variety of certification labels, which leads to a level of resistance to the perception of certification [25,59,60]. For instance, in France, the majority of national chocolate brands have one or more cocoa sustainability labels, including the organic label (Agriculture Biologique), the Fairtrade label (Max Havelaar), the Rainforest label, the Cocoa Plan label, the Cocoa Life label, the Carbon Neutral Product label, the UTZ label and the Palm Oil Free label. Other survey results [10] show that respondents support the effects of marketing communication through certification, but their expectations are higher than what they experience. As such, consumer skepticism influences buying behavior and its relationships with other antecedents [60,61]. The widespread use of food labels of various marketing terms referring to aspects of marketable food quality, e.g., “traditional”, “artisanal”, “natural”, “just like grandma’s”, “hand-made”, etc., is also a reason for the limited consumer confidence in this communication tool. Such attractive inscriptions or graphics used on food packaging give the impression of quality but have little or no connection with the production process [62].

In the process of transforming food systems towards sustainability, our research results can be used in developing strategies to increase knowledge about Food Quality Assurance Schemes (FQAS) and B2C certification. The national and local governments in both cases should finance the implementation of such strategies in order to build trust in certified food. In Poland, the strategies should apply mainly to international and EU certificates; in Belgium, in contrast, they should be applied to national certificates. Belgium is an example of a country that has been successful in promoting Fairtrade food, through a multi-faceted and multi-tool campaign funded by the budget of the Region of Flanders [47]. This explains why respondents from Belgium trusted this certificate the most, followed by the Euro Leaf certificate, as with Polish respondents. Despite the “Three Flavour Marks” campaign, their recognition among Polish respondents was very low. Campaigns promoting organic food are more often organized on a local scale (festivals, fairs, etc.) and on a national scale. Since 2022, the “Switch to Eco—Look for the Euro Leaf” campaign has been running in Poland, aiming to promote food labeled with this EU organic food certificate. This may be the reason that the Euro Leaf was considered trustworthy by Polish respondents in our research. Trust and transparency in any labeling scheme is essential for it to be meaningful and motivate change in individuals or industries. However, there has been confusion with regard to using the term “organic” on food products [37]. These products can be associated with what is known as a health halo. Assigning a health attribute to organic products is probably more related to the values attached to organic production practices than to the food itself, as there has been limited evidence to suggest the higher nutritional quality of organic products [37,63]. It seems justified to include an unambiguous certificate regarding the suitability of food products for sustainable food consumption in the EU’s pro-environmental strategies. The results of the survey reported in Special Eurobarometer 505 [41] indicate that clear information regarding the product’s environmental, health and social impacts would help 41% of respondents in the EU to adopt a sustainable diet. This was confirmed by the higher proportion of Belgian respondents (44%) compared to the much lower proportion (26%) of Polish respondents. Similar responses were recorded for the idea of the compulsory labeling of food sustainability. This was affirmed by 49% of respondents (on average in all studied EU countries), and by 55% of Belgians and 40% of Poles. Research by the European Consumer Organisation (BEUC) [64] also showed support for this solution, as most consumers (57%) from 11 EU Member States wished for sustainability information to be compulsory on food products. Almost half (47%) of Belgians agreed with this idea. This finding is in line with the responses, where a lack of clear labeling was revealed as one of the main perceived barriers to sustainable eating.

The current sustainability labeling landscape in the EU faces the challenge of labels being too numerous, too complex and too similar and with ambiguous information. Sustainability certification is also perceived to be insufficiently supportive of consumers committed to sustainability. Several proposals for a single labeling scheme have been described by various organizations or governments, with graphic symbols—meta-labels—linking the various dimensions of sustainability and communicating the overall sustainability performance of the product to consumers [17]. The European Union will make it mandatory for companies to use the Product Environmental Footprint (PEF) method when labeling products with environmental claims. PEF is a harmonized EU Life Cycle Assessment (LCA) methodology and covers sixteen life cycle impacts, including climate change, water consumption and the depletion of natural resources [65]. In the meantime, an increasing number of environmental labels are entering the market—for example, in Denmark, the Climate Score; in Switzerland, the Eaternity Score; in the UK, the Sustainability Scoring Label or Eco-Score; and in France, the Planet-Score label or the Eco Impact labeling scheme developed by Foundation Earth [66,67,68]. The influence of such interpretative labels can be enhanced by providing information to consumers [69].

Developing a single standard for environmental impact labeling in the European market is a challenge, even in the framework of the F2F strategy. A single label will require a huge investment in information and promotion campaigns, as with all activities in the free choice market. A multi-criteria system for sustainable food labeling needs to be created and agreed upon in all Member States, but setting some standards, like biodiversity, can be a hurdle [70]. However, even the most informed and environmentally conscious consumer cannot continue to be the sole focus in terms of increasing the number of sustainable food products purchased. It is essential to align the market offer with sustainable purchasing, including merchandising activities and effective promotion [71]. The need for producers to be more aware of the need to invest in the communication activities in order to “declare” (i.e., make more explicit) and substantiate (i.e., make more credible and transparent) their commitment towards socio-environmental issues was recently highlighted in a study of young Italian consumers [61].

### 4.1. Limitations and Strengths

The results of our study should be considered in the context of the participants’ attributes, which were primarily due to the method of sampling. Based on the authors’ knowledge of the determinants of purchasing behavior and the structure of food consumption in both countries, it was deliberately decided to carry out the survey among consumers interested in high-quality food. The choice of the two European capitals and of special food events as the locations for the quantitative study probably impacted certain sociodemographic characteristics of the respondents and their answers. On the other hand, this guaranteed the collection of an adequate sample size of 659 respondents. The deliberate choice of survey sites and sample size do not allow the generalization of the results. However, they provide valuable information about consumers’ views on the issue of sustainable food labeling and can be helpful in developing educational and awareness campaigns.

### 4.2. Strengths and Original Contributions

The main strength and original contribution of our study was the capturing of differences and similarities between respondents from two diverse—both culturally and economically—EU countries: one “old” Member State, i.e., Belgium, and a fairly “new” one, i.e., Poland. While sustainability is a global phenomenon, cross-cultural and regional factors may influence consumers’ selection of sustainable products, including differences in cultural values, environmental and social priorities, traditions, government roles and stages of economic development [72].

By combining qualitative and quantitative data, we have advanced the research on the perceptions of food certification and provided insights for policymakers and other food system stakeholders, highlighting the need to educate and reassure consumers that trustworthy certifications can help them to make more sustainable purchasing decisions.

## 5. Conclusions

In order for consumers to make more sustainable purchasing decisions on the food market, it is essential that they become aware of the environmental, social and economical costs of the food that they buy. Food certification is an important communication tool between the producer/supplier/retailer and the purchaser, so its informational and educational role cannot be overestimated. However, our research has shown the following:

1. Urban residents of two European capital cities—Warsaw and Brussels—are quite conservative in terms of their purchasing behavior and point to the freshness of products as its most important determinant.
2. Certificates confirming the exceptional quality of products or the production process were found to be moderately important factors in food choices. Brussels residents appeared to be more conscious and therefore more responsible food buyers. They took sustainability certifications into account in their purchasing decisions to a slightly greater extent. A larger share of Belgian respondents believed that certified food was of better quality and declared the significantly higher visibility of such foods and trust in certificates. This was especially true for international or EU certificates.
3. Warsaw residents were more skeptical about the labeling of food products with sustainable certificates and their trust was lower than that of Belgian respondents. In addition, in the trust ranking, the five highest marks were awarded by Belgian respondents to international certificates, while Polish respondents awarded them to national certificates, and the only exception was the Euro Leaf certificate.

It should be noted that improving the impact of food certification in the EU is a complex and constantly evolving issue and requires a comprehensive approach that includes government policy, industry practices and consumer awareness. Based on our study findings, food certification policies need to set clear and comprehensive standards for the labeling and use of sustainability certificates in the food industry. Collaboration between governments, NGOs, companies and certification bodies should be regulated to develop common sustainability standards and frameworks. It is also necessary to educate consumers about the importance of sustainability and increase their confidence in certifications by organizing educational campaigns, workshops or events. To increase the effectiveness of sustainability certification, it is also necessary to provide consumers with tools and services, including smartphone apps or QR codes for instant access to the certification details.

In summary, certification labels have a limited impact on consumers’ decisions, even if they understand them and are generally interested in environmental, social and ethical issues related to food. This situation is due to the large number of certificates and certified foods (leading to consumer confusion), the lack of uniformity of labels (making it difficult for them to compare products effectively), greenwashing, the limited visibility and availability at the point of sale, the price sensitivity of consumers and the prioritization of other factors. To overcome these limitations, it is important that food certification labels are standardized, well-regulated and accompanied by education and awareness campaigns. Companies should also integrate sustainability into their core values and practices, rather than using labels as a marketing gimmick. Ultimately, increasing consumer awareness and making sustainable choices more accessible and affordable could help to increase the impact of sustainability labels and boost the demand for certified foods in the region.

## Figures and Tables

**Table 1:** Sociodemographic characteristics of the quantitative study sample.

Variable			Total Sample (%) *n* = 659	Poland (%) *n* = 330	Belgium (%) *n* = 329
Gender	Male		36.9	38.9	35.0
	Female		63.1	61.1	65.0
Age (years)	Below 25		22.2	29.9	16.7
	26–35		33.7	19.3	37.6
	36–45		19.0	12.5	18.6
	46–55		12.6	10.9	12.8
	Over 55		12.6	27.4	14.3
Education	Primary or vocational		6.7	5.4	7.9
	Secondary		35.2	19.2	18.1
	Higher		58.1	75.4	74.0
Household size (number of people)	1		19.6	13.1	26.4
	2		33.9	29.4	38.6
	3		18.3	21.3	15.2
	4 and more		28.3	36.2	19.8
Household average monthly income * (per person)	PLN	EUR			
	<1000	-	4.2	8.4	-
	1001–1500	<1500	22.7	17.9	28.0
	1501–2000	1501–2500	30.8	17.6	45.3
	2001–3000	2501–3000	19.1	23.2	14.5
	3001–4000	3501–4500	9.7	12.2	6.9
	>4000	>4500	13.3	20.7	5.3
Household financial situation (subjective assessment)		Very good	12.5	13.5	11.5
		Rather good	42.7	40.8	44.8
		Average	37.9	39.6	36.1
		Rather bad	5.2	4.9	5.4
		Very bad	1.7	1.2	2.2
		No	22.7	25.3	19.8

* Considerable differences in the nominal income levels of the respondent groups are due to differences in average wages in both countries, price structures and exchange rates (PLN and EUR).

**Table 2:** Perception of the certified product as being better than others in the same category, % of respondents.

	% of Respondents			
	Total Sample *(n* = 659)	Warsaw Residents *(n* = 330)	Brussels Residents *(n* = 329)	*p*-Value
Yes	38	33	44	
No	41	49	31	0.0001
I don’t know	21	18	25	

**Table 3:** The visibility of products bearing international or EU certificates (5-point scale, where 1—not available, 5—highly available at points of sale).

Certification Label	Order *	Warsaw Residents	Brussels Residents
Euro Leaf	PL 7, BE 5	3.06 ^b,1^	3.41 ^a^
Protected Designation of Origin—PDO	PL 11, BE 7	2.61 ^b^	3.09 ^a,3^
Fairtrade	PL 12, BE 2	2.59 ^b,2^	3.68 ^a,2^
Protected Geographical Indication—PGI	PL 14, BE 8	2.53 ^b^	3.07 ^a^
Traditional Speciality Guaranteed—TSG	PL 15, BE 13	2.51	2.80
Rainforest Alliance Certified	PL 16, BE 9	2.47	3.00
Certified Sustainable Seafood—MSC	PL 17, BE 11	2.43	2.97
Slow Food	PL 18, BE 15	2.35	2.70

* indicates the order in which products with a given certificate are visible at the point of sale in a given country: PL—Poland and BE—Belgium. Superscript letters indicate whether Polish and Belgian assessments differ significantly for each label (*p* ≤ 0.05). Numbers indicate relationships between the visibility of a given certificate and respondents’ sociodemographic characteristics (Polish or Belgian); 1—age, 2—education, 3—household size.

**Table 4:** The trust level in international or EU certificates (5-point scale, where 1—untrustworthy, 5—very trustworthy).

Certification Label	Order *	Warsaw Residents	Brussels Residents
Euro Leaf	PL 2, BE 2	3.59	3.75
Traditional Speciality Guaranteed—TSG	PL 12, BE 14	2.88	2.70
Protected Geographical Indication—PGI	PL 13, BE 13	2.87 ^1,2^	2.97
Fairtrade	PL 14, BE 1	2.73 ^b^	3.89 ^a^
Protected Designation of Origin—PDO	PL 15, BE 8	2.72 ^2^	3.25
Slow Food	PL 16, BE 4	2.64 ^b^	3.45 ^a,3^
Certified Sustainable Seafood—MSC	PL 17, BE 3	2.62 ^b^	3.47 ^a^
Rainforest Alliance Certified	PL 18, BE 5	2.46 ^b,3^	3.37 ^a^

* indicates the order in which products with a given certificate fall into the trust ranking in a given country: PL—Poland and BE—Belgium. Superscript letters indicate whether Polish and Belgian perceptions differ significantly (*p* ≤ 0.05). Numbers indicate relationships between the trust in a given certificate and respondents’ sociodemographic characteristics (Polish or Belgian); 1—age, 2—education, 3—household size.

**Table 5:** Impact of the certificate logo on the decision to purchase food, % of respondents.

		% of Respondents		
Answer	Total Sample *n* = 659	Warsaw Residents *n* = 330	Brussels Residents *n* = 329	*p*-Value
Yes	26	24	28	
It depends on the products’ type	52	53	51	
No	19	19	18	0.712
I am not able to tell	3	4	3	

**Table 6:** Comparison of the impact of certificate logo when choosing food for focus group participants, depending on gender and place of residence.

	Warsaw Residents	Brussels Residents
Female	***	****
	Rather a positive impact on purchases; it is better if the package has a certificate label than not, i.e., *“I’m always looking for certificates and I’m very happy to see if there’s a mark because it means that someone has leaned over this product, that the company itself is sure that the product is worth something”.*	A positive impact on purchases, actively looking for certificate label on product packaging during purchases, i.e., *“I more often pay attention to Fairtrade, Rainforest Alliance Certified, Sustainable Fishing, because I am increasingly concerned about the issues of fisheries, the way of production and environmental impact, as well as social justice”.*
Male	*	**
	Limited confidence, weak influence on purchasing decisions, i.e., *“There are many of these certificates, maybe my knowledge is too small, but I don’t trust them, mainly because there are so many of them and they don’t give me any specific guarantee”.*	Limited confidence, moderate influence on purchasing decisions, i.e., *“When I see too many marks on a product, it makes me suspicious, I have the impression that the manufacturer is trying to push me the product”.*

Influence of certificates: **** major effect; *** moderate effect, ** neutral, * minor effect.

**Table 7:** Mean evaluation scores of factors influencing consumer food choice.

Factors	Total Sample *n* = 659	Warsaw Residents *n* = 330	Brussels Residents *n* = 329
Freshness	4.62	4.74 ^a^	4.49 ^b,2^
Ingredients	3.84	3.84 ^1,2,3^	3.84 ^1^
Price	3.52	3.55 ^4,5^	3.49 ^3^
Information on the packaging, e.g., no sugar, no preservatives, natural, etc.	3.37	3.35 ^1^	3.39 ^5^
Nutritional and caloric value	3.42	3.62 ^a,5^	3.20 ^b,5^
Brand reputation, trust towards the producer	3.30	3.47 ^a^	3.13 ^b,2^
Symbols or certificates on the packaging indicating special qualities	3.10	3.04	3.16 ^4^
Symbols on the packaging that indicate a distinction of a product in a competition, an award, etc.	2.61	2.79 ^a^	2.42 ^b,2,3,5^
Practical, convenient packaging	2.59	2.83 ^a,1^	2.33 ^b,3,5^
Promotion (tasting, gifts, etc.)	2.48	2.53 ^1,2^	2.42 ^1,2^
Esthetical packaging	2.41	2.75 ^a^	2.05 ^b,3^
Advertising	2.06	2.17 ^a,2,4^	1.94 ^b,2^

Superscript letters indicate whether Polish and Belgian assessments differ significantly for each factor (*p* ≤ 0.05). Numbers indicate relationships between the particular factor and respondents’ sociodemographic characteristics (Polish or Belgian); 1—age, 2—education, 3—household size, 4—household average monthly income, 5—household financial situation.

**Table 8:** The importance of food choice factors for focus group participants.

Factors	Warsaw Residents		Brussels Residents	
	Female	Male	Female	Male
Sensory attributes: taste, appearance, etc.	4	5	5	4
Product freshness and its shelf life	5	3	5	4
Brand reputation, trust towards the producer	4	3	5	5
Product composition and nutritive value	5	3	5	2 if it is bought for the first time
Price	3	4	2 in reference to quality	3
Packaging	3	2 innovative solutions, eye-catching	4	4
Country and place of origin	4	2 ethnocentric attitudes	5	3
Promotion, advertising	3	3	3	3
Quality labels, symbols and certificates	2, whether present on packaging or not	1	3	1
Nutrition and health claims	3	2 product composition	1	1
Season	2	1	3	1

5—very important; 4—important; 3—rather important; 2—considered in relation to another product attribute; 1—unaffected/irrelevant.

## Data Availability

Data are not publicly available, although the data may be made available on request to the corresponding author.

## Appendix A. Detailed Results of the Analysis

**Table A1:** Correlations between the importance of food choice factor ratings and the sociodemographic characteristics of the respondents (Cramer’s V test).

Factors	Warsaw Residents *n* = 330		Brussels Residents *n* = 329	
Freshness	ns *		education	*p* = 0.0003 V = 0.2020
Ingredients	age	*p* = 0.0269 V = 0.1516	age	*p* = 0.0025 V = 0.1771
	education	*p* = 0.0043 V = 0.1651		
	household size	*p* = 0.0090 V = 0.1868		
Price	household average monthly income	*p* < 0.0001 V = 0.2244	household size	*p* < 0.0001 V = 0.2506
	household financial situation	*p* = 0.0001 V = 0.1890		
Information on the packaging, e.g., no sugar, no preservatives, natural, etc.	age	*p* = 0.0140 V = 0.1563	household financial situation	*p* = 0.0441 V = 0.1501
Nutritional and caloric value	household financial situation	*p* = 0.0002 V = 0.1867	household financial situation	*p* = 0.0076 V = 0.1687
Brand reputation, trust towards the producer	ns		education	*p* = 0.0250 V = 0.1613
Symbols or certificates on the packaging indicating special qualities	ns		household average monthly income	*p* = 0.0487 V = 0.1549
Symbols on the packaging that indicate the distinction of a product in a competition, an award, etc.	ns		education	*p* = 0.0389 V = 0.1557
			household size	*p* = 0.0036 V = 0.1731
			household financial situation	*p* = 0.0036 V = 0.1731
Practical, convenient packaging	age	*p* = 0.0488 V = 0.1514	household size	*p* = 0.0056 V = 0.2125
			household financial situation	*p* = 0.0386 V = 0.1547
Promotion (e.g., tasting, gifts, etc.)	age	*p* = 0.0164 V = 0.1569	age	*p* = 0.0488 V = 0.1514
	education	*p* = 0.0142 V = 0.1561	education	*p* = 0.0001 V = 0.1951
			household financial situation	*p* = 0.0012 V = 0.1820
Esthetical packaging	ns		household size	*p* = 0.0010 V = 0.2231
Advertising	age	*p* = 0.0377 V = 0.1471	education	*p* = 0.0007 V = 0.1976
	household average monthly income	*p* = 0.0129 V = 0.1728		

* ns—not statistically significant.

**Table A2:** Correlations between the perception of the visibility of products with a specific certification label and the sociodemographic characteristics of the respondents (Cramer’s V test).

Certification Label *	Warsaw Residents		Brussels Residents	
1 (PL) Laur Konsumenta	age education	*p* = 0.0005 V = 0.2032 *p* = 0.0029 V = 0. 1882	ns **	
2 Euro Leaf	age	*p* = 0.0035 V = 0.2322	ns	
3	ns		ns	
4 Protected Designation of Origin (PDO)	ns		household size	*p* = 0.0273 V = 0.2812
5	ns		ns	
6	ns		ns	
7 (BE) La Bleue des Prés	ns		household financial situation	*p* = 0.0398 V = 0.4120
8 (PL) Poznaj Dobrą Żywność	age	*p* = 0.0274 V = 0.1893	ns	
9	ns		ns	
10 (PL) Integrowana Produkcja; (BE) Les Fromages de chez nous/Kazen van bij ons	household average monthly income	*p* = 0.0017 V = 0.3716	education	*p* = 0.0404 V = 0.2300
11	ns		ns	
12	ns		ns	
13	ns		ns	
14	ns		ns	
15	ns		ns	
16 Fairtrade	education	*p* = 0.0378 V = 0.2628	education	*p* = 0.0005 V = 0.2535
17	ns		ns	
18	ns		ns	
19 (BE) Agriculture de Wallonie	ns		household average monthly income	*p* = 0.0270 V = 0.3315
20	-		ns	

* 1—PL: Laur Konsumenta/BE: Prix Monde Selection, 2—Euro Leaf, 3—PL: Teraz Polska/BE: Ambao, 4—Protected Designation of Origin PDO, 5—Protected Geographical Indication PGI, 6—Traditional Specialty Guaranteed TSG, 7—PL: Gwarantowana Jakość/BE: La Bleue des Prés, 8—PL: Poznaj Dobrą Żywność/BE: Best Frit, 9—PL: Znak Jakości Q/BE: Responsibly Fresh, 10—PL: Integrowana Produkcja/BE: Les Fromages de chez nous/Kazen van bij ons, 11—PL: Quality Meat Program QMP/BE: BCV, 12—PL: Pork Quality System PQS/BE: Certus, 13—PL: System Gwarantowanej Jakości Żywnośći/BE: Meesterlyck/Magistral, 14—PL: Jakość Tradycja/BE: Streekproduct/Regio&Traditie, 15—Slow Food, 16—Fairtrade, 17—Rainforest Alliance Certified, 18—Certified Sustainable Seafood MSC, 19—PL: Agro Polska/BE: Agriculture de Wallonie, 20—BE: Flandria. ** ns—not statistically significant

**Table A3:** Correlations between consumers’ trust in a specific certification label and the sociodemographic characteristics of the respondents (Cramer’s V test).

Certification Label *	Warsaw Residents		Brussels Residents	
1 (PL) Laur Konsumenta	household average monthly income	*p* = 0.0396 V = 0.1774	ns **	
2	ns		ns	
3 (BE) Ambao	ns		household financial situation	*p* = 0.0159 V = 0.3940
4 Protected Designation of Origin (PDO)	education	*p* = 0.0194 V = 0.2713	ns	
5 Protected Geographical Indication (PGI)	age education	*p* = 0.0403 V = 0.2578 *p* = 0.0164 V = 0.2087	ns	
6	ns		ns	
7	ns		ns	
8	ns		ns	
9 (PL) Znak Jakości Q; (BE) Responsibly Fresh	age	*p* = 0.0226 V = 0.1801	household average monthly income	*p* = 0.0485 V = 0.4095
10 (PL) Integrowana Produkcja; (BE) Les Fromages de chez nous/Kazen van bij ons	household average monthly income	*p* = 0.0322 V = 0.3304	age	*p* = 0.0017 V = 0.2567
11 (BE) BCV	ns		age	*p* = 0.0425 V = 0.3562
12	ns		ns	
13	ns		ns	
14	ns		ns	
15 Slow Food	ns		household size	*p* = 0.0320 V = 0.3193
16	ns		ns	
17 Rainforest Alliance Certified	household size	*p* = 0.0348 V = 0.3246	ns	
18	ns		ns	
19	ns		ns	
20	-		ns	

* 1—PL: Laur Konsumenta/BE: Prix Monde Selection, 2—Euro Leaf, 3—PL: Teraz Polska/BE: Ambao, 4—Protected Designation of Origin PDO, 5—Protected Geographical Indication PGI, 6—Traditional Specialty Guaranteed TSG, 7—PL: Gwarantowana Jakość/BE: La Bleue des Prés, 8—PL: Poznaj Dobrą Żywność/BE: Best Frit, 9—PL: Znak Jakości Q/BE: Responsibly Fresh, 10—PL: Integrowana Produkcja/BE: Les Fromages de chez nous/Kazen van bij ons, 11—PL: Quality Meat Program QMP/BE: BCV, 12—PL: Pork Quality System PQS/BE: Certus, 13—PL: System Gwarantowanej Jakości Żywnośći/BE: Meesterlyck/Magistral, 14—PL: Jakość Tradycja/BE: Streekproduct/Regio&Traditie, 15—Slow Food, 16—Fairtrade, 17—Rainforest Alliance Certified, 18—Certified Sustainable Seafood MSC, 19—PL: Agro Polska/BE: Agriculture de Wallonie, 20—BE: Flandria. ** ns—not statistically significant.

## Appendix B. Quantitative Research Questions Analyzed in This Article

**Table A4:** Quantitative research questions analyzed in the article.

**Q1. Please mark on ‘1 to 5 scale’ how important are for you the following factors influencing your choice of food products, where 1—least important, 5—most important** *Single-response matrix question with a five-level ascending scale*					
Factors	1—least important	2	3	4	5—most important
Price					
Ingredients					
Symbols or certificates on the packaging indicating special quality					
Freshness					
Nutritional and caloric value					
Esthetical packaging					
Practical, convenient packaging					
Brand reputation, trust towards the producer					
Promotion (e.g., tasting, gifts, etc.)					
Advertising					
Information on the packaging, e.g., no sugar, no preservatives, natural, etc.					
Symbols on the packaging that indicate the distinction of a product in a competition, an award, etc.					
**Q2. Do you think that a product marked with a symbol or certificate on the packaging is a better one that others in the same category?** *Single choice question*					
	Yes—why? No I don’t know				
**Q3. (visibility) Do you see certified food products at points of sale with the symbols below? Please mark on ‘1 to 5 scale’, where 1—not available, 5—highly available at points of sale** *Single-response matrix question with a five-level ascending scale*					
**Q4. (trust) Do you trust in particular certified labels?** **Please mark on ‘1 to 5 scale’, where 1—untrustworthy and 5—very trustworthy** *Single-response matrix question with a five-level ascending scale*					
Name and graphic logo of certificate	1—not available/ untrustworthy	2	3	4	5—highly available/very trustworthy
Laur Konsumenta (PL) or Prix Monde Selection (BE)					
Euro Leaf (PL + BE)					
Teraz Polska (PL) or Ambao (BE)					
Protected Designation of Origin PDO (PL + BE)					
Protected Geographical Indication PGI (PL + BE)					
Traditional Specialty Guaranteed TSG (PL + BE)					
Gwarantowana Jakość (PL) or La Bleue des Prés (BE)					
Poznaj Dobrą Żywność (PL) or Best Frit (BE)					
Znak Jakości Q (PL) or Responsibly fresh (BE)					
Integrowana produkcja (PL) or Les fromages de chez nous/Kazen van bij Ons. (BE)					
Quality Meat Program (PL) or BCV(BE)					
Pork Quality System PQS (PL) or Certus (BE)					
System Gwarantowanej Jakości Żywności (PL) or Meesterlyck/Magistral (BE)					
Jakość Tradycja (PL) or Regio & Traditie (BE)					
Slow food (PL + BE)					
Fairtrade (PL + BE)					
Rainforest Alliance Certified (PL + BE)					
Certified Sustainable Seafood MSC (PL + BE)					
Agro Polska (PL) or Agriculture de Wallonie (BE)					
Flandria (BE)					
**Q5. Does the presence of certification symbols on food packaging influence your purchasing choice?** *Single choice question*					
	Yes It depends on the products’ type No I am not able to tell				
